# Same-day Discharge After Minimally Invasive Transforaminal Lumbar Interbody Fusion: A Series of 808 Cases

**DOI:** 10.1007/s11999-013-3366-z

**Published:** 2013-11-23

**Authors:** Walter W. Eckman, Lynda Hester, Michelle McMillen

**Affiliations:** Aurora Spine Center, PO Box 3660, Tupelo, MS 38803 USA

## Abstract

**Background:**

The versatility of transforaminal lumbar interbody fusion (TLIF) allows fusion at any level along with any necessary canal decompression. Unilateral TLIF with a single interbody device and unilateral pedicle fixation has proven effective, and minimally invasive techniques have shortened hospital stays. Reasonable questions have been raised, though, about whether same-day discharge is feasible and safe after TLIF surgery.

**Questions/purposes:**

We determined, in a high-volume spine practice, what proportion of patients having one- or two-level minimally invasive unilateral TLIF go home on the day of surgery or stay longer and compared the two groups in terms of outcome scores (VAS scores for back and leg pain, Waddell-Main Disability Index), complications, and hospital readmissions.

**Methods:**

We retrospectively studied all 1005 patients who underwent 1114 minimally invasive unilateral TLIF procedures by one surgeon between March 18, 2003, and April 12, 2013. For the first 43 months, Medicare patients (65 years or older) were not offered same-day discharge. All other patients were offered the chance to be discharged home on the same day if they felt well enough. Followup data were for 3 months. VAS scores for back and leg pain and Waddell-Main Disability Index were recorded in a prospectively maintained database and readmissions were ascertained by chart review. Data were available on 100% of discharges, 95% of preoperative outcome scores, and 81% of outcome scores out to 3 months.

**Results:**

Of the 1114 procedures, 808 went home the day of surgery, resulting in a 73% same-day discharge rate. Mean differences in outcome scores from preoperatively to 3 months were similar between groups, except for a difference in VAS lower leg pain in hospital stay patients, which was of borderline statistical and unlikely clinical significance (3.3 versus 2.7, p = 0.05). The only important differences between groups were slightly more medical complications and readmissions for patients 65 years and older who stayed in hospital overnight (3.9% versus 0%, p < 0.01); however, some self-selection bias toward staying overnight among patients with higher self-rated disability and pain scores likely accounted for this difference.

**Conclusions:**

Surgeons experienced in minimally invasive spine surgery can consider same-day discharge for patients having minimally invasive unilateral TLIF procedures.

**Level of Evidence:**

Level III, therapeutic study. See Instructions for Authors for a complete description of levels of evidence.

## Introduction

The goal of minimally invasive spine surgery is to minimize soft tissue disruption and reduce blood loss, pain, and hospital stay while speeding patient recovery. While there is some controversy on the topic, some research demonstrates the efficacy of minimally invasive approaches for numerous conditions affecting the lumbar spine [13].

Transforaminal lumbar interbody fusions (TLIFs) have been simplified with unilateral procedures. Use of a single interbody device [1, 4, 6, 7, 9, 14, 16–18] and unilateral pedicle screw fixation [1, 4, 5, 9, 14, 16, 18] has likewise proven effective. These modifications make less invasive approaches more feasible and, by making the surgical procedures less traumatic, may facilitate same-day discharge. However, we found no published series of same-day discharge after lumbar fusion.

We therefore evaluated whether patients could safely go home on the day of surgery if given the choice by determining same-day discharge rate, clinical outcomes (VAS scores for back and leg pain, Waddell-Main Disability Index), complications, and hospital readmissions in a series of patients of all ages undergoing one- or two-level minimally invasive unilateral TLIF with a single interbody device per level and unilateral fixation.

## Patients and Methods

### Study Design and Patient Selection

This was a retrospective study, which was approved by the institutional review board of the host center. Between March 18, 2003, and April 12, 2013, the senior author (WWE) performed 1134 one- or two-level lumbar fusions of which 1114 (98.2%) were minimally invasive unilateral TLIF procedures forming the series for this study. Twenty procedures (1.8%) were bilateral minimally invasive TLIFs and were omitted from this study. There were no traditional open lumbar fusions or other approaches. Unilateral procedures were divided into two unmatched cohorts for comparative analysis: same-day discharge or hospital stay. This study focused on discharge day and early clinical results out to 3 months. Discharge date was available for 100% of procedures. Clinical outcome data were recorded for 95% at baseline and 81% through 3-month followup. Discharge date, medical readmissions, and comorbidities were available from hospital records, and scores for function and pain were all obtained from patients at time of service. Data collection was limited in the first few months of this study. Subsequently, data were obtained for Waddell-Main Disability Index and VAS scores for back and leg pain. For the first 43 months, same-day discharge was not encouraged for patients 65 years or older due to Medicare regulations requiring admission and uncertainty about possible need for longer observation. An additional 14 patients were not discharged early because they had more severe loss of function or surgical or medical complications; these patients were included in the series in the hospital stay group, though not all of these patients were older than 65 years. Otherwise, all patients were offered the choice to go home or stay in hospital. There were no other selection or exclusion criteria.

### Patient Population

The same-day discharge cohort consisted of 728 patients (376 male, 352 female) who had 808 procedures at 862 levels (54 at two levels). Mean age was 52 years (range, 13–86 years). The hospital stay cohort included 277 patients (112 male, 165 female) who had 306 procedures at 339 levels (33 at two levels). Mean age was 64 years (range, 15–89 years). Because the groups were self-selecting, they were not comparable in age, baseline activity, pain, or medical complexity. The hospital stay cohort was older (p < 0.001) and had higher preoperative back pain scores (p = 0.015), but preoperative Waddell-Main Disability Index scores were not different between groups (p = 0.189). All patients had chronic back and/or leg pain. Almost all had multiple diagnoses of lumbar spine degenerative disorders in various combinations (Table 1). Differences can be partly explained based on age, with more stenosis in the older hospital stay group and more recurrent disk herniations in the younger same-day surgery group. As one would expect, the large majority of the patients had surgery in the lower lumbar spine (Table 2).

**Table 1:** Primary diagnosis categories in the two study groups

Diagnosis	Percentage of procedures		p value
	Same-day discharge group	Hospital stay group	
Stenosis			
Canal	26.67 ± 4.57	42.71 ± 6.87	< 0.001
Lateral recess/foraminal	15.0 ± 3.69	19.60 ± 5.50	0.192
Listhesis			
Ventral	14.72 ± 3.66	11.06 ± 4.36	0.245
Dorsal	7.22 ± 2.67	2.01 ± 1.95	0.001
Lateral/scoliosis	0.83 ± 0.94	0.50 ± 0.98	1.000
Segmental instability	4.72 ± 2.19	2.51 ± 2.17	0.258
Acute/recurrent disk herniation	17.78 ± 3.95	10.05 ± 4.18	0.014
Positive discography	13.06 ± 3.48	11.56 ± 4.44	0.689

Values are expressed as mean ± CI.

**Table 2:** Levels selected for fusion in the two study groups

Level	Number of procedures	
	Same-day discharge group	Hospital stay group
L1-L2	2 (0.23%)	3 (0.88%)
L2-L3	40 (4.64%)	24 (7.08%)
L3-L4	139 (16.13%)	71 (20.94%)
L4-L5	391 (45.36%)	179 (52.80%)
L5-L6	14 (1.62%)	3 (0.88%)
L5-S1	272 (31.55%)	59 (17.40%)
L6-S1	4 (0.46%)	0 (0%)
Total	862	339

### Power Analysis

Choosing an alpha value of 0.05 and a beta value of 0.20, sample size can be calculated when SD and effect size are known. Estimating a sample with a mean of 4 and an SD of 3.5 and a second sample with a mean of 3 and an SD of 2.0 would require a sample size of 131 for statistical significance. This study included dependent sample sizes from 245 to 625 with effect sizes (Cohen’s d) from 0.91 to 2.42, resulting in a level of significance of less than 0.001 and a statistical power of 100% for all outcomes in both cohorts.

### Surgery

**Fig. 1:**
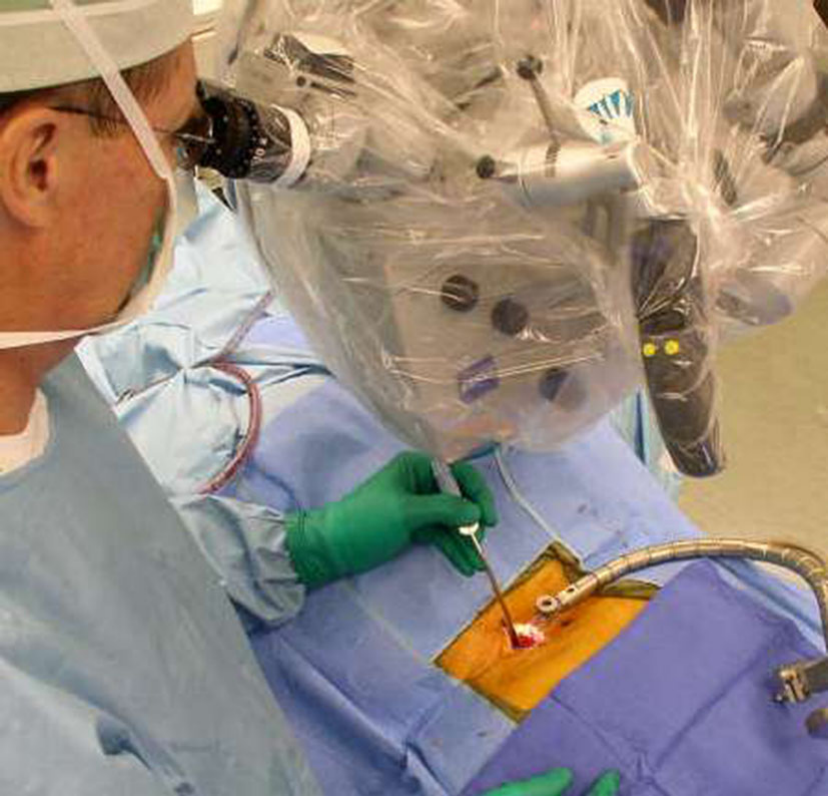
Unilateral minimally invasive TLIF including insertion of interbody device and unilateral pedicle screws is completely performed through a single incision using a small closed working channel.

All patients had minimally invasive unilateral TLIF through a single incision using a small closed working channel (21-mm diameter) avoiding muscle retraction (Fig. 1). All had a single interbody device per level (titanium 84%, polyetheretherketone 16%), interbody BMP-2 or silicate-substituted hydroxyapatite bone growth substitutes, and unilateral pedicle screw fixation. Eighty-six percent had additional unilateral posterior-lateral fusion.

### Postoperative Protocol

A thoracolumbar orthosis with sternal support was used for all patients for 12 weeks initially, which was later reduced to 6 weeks. Almost all patients were ambulatory within 2 hours. Radiographs were taken at 1 week and at 6 to 12 weeks (when the brace was removed). Followup included outcome evaluation through 3 months.

### Study End Points

Primary outcomes included discharge day and clinical scores for function and pain. Changes in functional status were evaluated using Waddell-Main Disability Index scores, which have been validated and correlated with the Oswestry Disability Index [12, 15]. Pain levels for the back and upper and lower legs were measured using a 10-point VAS, which has been validated for pain measurement [3, 10, 11]. Secondary measures were rates of transfusion, infection, return to work for patients working up to 30 days before surgery, repeat surgery at the index level, and major medical complications and readmissions within 2 weeks of surgery.

### Statistical Analysis

The same-day discharge sample was determined by patient choice with no selection process and no similar series for statistical comparison. For further analysis, the same-day discharge cohort was evenly divided into four groups of consecutive cases. Rates of same-day discharge over time were compared, with each later group compared to the first. Outcome scores, return to work rates, reoperations at index level, and medical complications and readmission rates were compared between groups. Outcome scores were compared between baseline and 3-month values by difference between means with dependent samples. This required individual scores at both time intervals for each case, with a slightly lowered total sample number for this analysis as a result. These mean differences for outcomes were then used to compare the same-day discharge and hospital stay groups by evaluating difference of means with independent samples.

Statistics of outcome scores are controversial since the results may be ordinal measures rather than interval or ratio measures [8], though there is evidence that the VAS is linear for pain [10]. The Waddell-Main Disability Index has shown essentially equal contribution from each of its nine items [15]; thus, the sums of any number of these items would be at regular intervals, and since there is a true zero, these scores appear to be ratio measures. Other clinical results have small values presented for information purposes. Level of significance was 0.05 for all CIs. All statistical analyses were by calculated effect sizes, CIs of proportions and CIs of means, and comparison of outcome means by Microsoft^®^ Excel^®^ (Microsoft Corp, Redmond, WA, USA). The Fisher’s exact test and T-test were used to measure level of significance. DSS Research software (Decision Support Systems, LP, Fort Worth, TX, USA) was used to calculate sample size and statistical power.

## Results

**Fig. 2:**
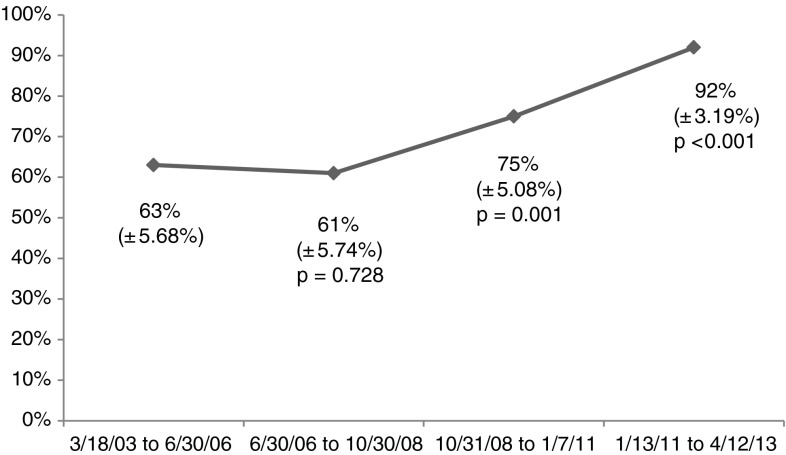
A graph shows that the rate of same-day discharge increased over the 10-year study period.

Of the 1114 procedures, 808 went home the day of surgery while 306 stayed in hospital, resulting in a 73% overall same-day discharge rate. The rate of same-day discharge increased over the 10-year study period (Fig. 2).

Scores for function and pain improved in both groups, with no differences between them (Table 3), other than a difference in VAS score for lower leg pain in hospital stay patients, which was of borderline statistical and unlikely clinical significance (3.3 versus 2.7, p = 0.05).

**Table 3:** Scores for function and pain in the two study groups

	SAME-DAY DISCHARGE OUTCOMES				HOSPITAL STAY OUTCOMES			
	Waddell-Main Disability Index	VAS Back Pain	VAS Upper Leg Pain	VAS Lower Leg Pain	Waddell-Main Disability Index	VAS Back Pain	VAS Upper Leg Pain	VAS Lower Leg Pain
Pre-op	5.17 (±.12)	6.67 (±.14)	5.01 (±.23)	3.70 (±.26)	5.30 (1 ± .18)	7.01 (±.23)	5.39 (±.37)	4.17 (±.42)
N	756	712	712	712	302	288	288	288
SD	1.67	1.97	3.19	3.57	1.57	2.0	3.22	3.67
3 Month	2.70 (±.19)	2.07 (±.15)	0.92 (±.14)	1.04 (±.16)	2.68 (±.20)	2.10 (±.24)	0.88 (±.23)	0.94 (±.24)
N	647	646	646	646	252	252	252	252
SD	2.49	1.98	1.80	2.11	1.59	1.97	1.86	1.97
Mean Difference	2.54 (±.17)	4.62 (±.21)	4.16 (±.27)	2.69 (±.31)	2.63 (±.27)	4.84 (±.32)	4.44 (±.47)	3.28 (±.49)
N (Dependent Sample)	625	618	618	618	248	245	245	245
SD	2.22	2.68	3.44	3.92	2.17	2.57	3.69	3.94
Effect Size (Cohen’s d)	1.49	2.34	1.61	0.91	1.64	2.43	1.70	1.12
p Value of Difference	<.001	<.001	<.001	<.001	<.001	<.001	<.001	<.001

	COMPARISON BETWEEN SAME DAY DISCHARGE AND HOSPITAL STAY			
	Waddell-Main Disability Index	VAS Back Pain	VAS Upper Leg Pain	VAS Lower Leg Pain
p Value Pre-op	0.189	0.015	0.098	0.069
p Value Mean Difference	0.629	0.259	0.302	0.050

In general, complications and readmissions were comparable in the two study groups. Transfusions usually required longer hospital stays and thus were almost all in the hospital stay cohort (Table 4). There were no differences in reoperations at the index level between groups (Table 5). Comparing patients with same-day discharge to those who stayed only one night in the hospital, the aggregate end point of complications plus readmissions was not different for patients younger than 65 years, but among patients 65 years and older, those who stayed in the hospital overnight had a higher likelihood of complications and readmission than those who went home the same day (3.9% versus 0%, p < 0.01; Table 6); however, some self-selection bias toward more disabled patients staying overnight likely accounted for this difference.

**Table 4:** Secondary outcomes in the two study groups

Outcome	Percentage of procedures		p value
	Same-day discharge group	Hospital stay group	
Transfusions	0.25 ± 0.34	1.63 ± 1.42	0.019
Infections	0.12 ± 0.24	0	1.000
Return to work	96.0 ± 1.35	93.0 ± 2.86	0.265
Reoperations	2.35 ± 1.04	4.58 ± 2.34	0.072

Values are expressed as mean ± CI.

**Table 5:** Reasons for early and late reoperations in the two study groups

Reoperation	Number of reoperations			
	Same-day discharge group		Hospital stay group	
	Early	Late	Early	Late
Symptomatic, explore fusion	0	4	0	0
Subsidence, stenosis, listhesis	3	1	0	2
Reexplore, decompression	1	0	2	0
Extradural hematoma/fluid	2	0	5	0
Revise screw/rod	1	0	4	0
Displaced fusion material	0	2	0	0
Nonunion	0	1	0	0
Superficial infection	0	1	0	0
Total	16		13

**Table 6:** Analysis of medical complications and readmissions in the two study groups

Variable	Same-day discharge group	Hospital stay group (1 night only)	p value
< 65 years old			
Early medical complications and readmissions (%)	0.94 ± 0.75 (n = 642)	0 (n = 101)	1.000
≥ 65 years old			
Age (years)	71.12 ± 0.77	72.21 ± 0.80	0.540
Early medical complications and readmissions (%)	0 (n = 166)	3.9 ± 3.03 (n = 157)	0.013
Waddell-Main Disability Index	4.58 ± 0.19 (n = 151)	5.05 ± 0.21 (n = 156)	0.001
VAS score for back pain (points)	6.61 ± 0.36 (n = 148)	6.97 ± 0.34 (n = 146)	0.156
Number of comorbidities	2.20 ± 0.22	1.71 ± 0.17	< 0.001

Values are expressed as mean ± CI.

## Discussion

It is important for surgeons and patients to know whether same-day discharge can be safe, reliable, and effective after lumbar fusion. Studies of minimally invasive spine surgery emphasize the goal of reducing hospital stay. However, limits to the feasibility and safety of early discharge after lumbar fusion have not been fully explored. We therefore evaluated whether patients could safely go home on the day of surgery if given the choice by determining same-day discharge rate, clinical outcomes (VAS scores for back and leg pain and Waddell-Main Disability Index), complications, and hospital readmissions in a series of patients of all ages undergoing one- or two-level minimally invasive unilateral TLIF with a single interbody device per level and unilateral fixation.

This study had several important limitations. Patient selection is biased by lack of choice for Medicare patients for the first 43 months (during which time Medicare regulations required admission) and by self-selection for other patients. The hospital stay group was older and included 14 patients with more serious medical and neurologic problems who had longer stays. There are missing data for preoperative scores (5%) and early postoperative disability and pain scores (19%). It is worth emphasizing that these are elective, scheduled spine procedures from an outpatient practice and that the patients were relatively healthy; no patients in the study were operated on as the result of an inpatient consultation, hospital transfer, or trauma. It also needs to be stated that the practice setting is specialized; with more than 100 nearly identical procedures a year, consistency of preoperative education, operative team performance, and postoperative nursing likely influenced our results, and surgical experience of the senior author includes more than 2000 minimally invasive lumbar spine surgeries. Results therefore may not generalize well to other practice settings.

In our series, same-day discharge was achieved in 808 of 1114 (73%) minimally invasive unilateral TLIFs. We have found no published study of same-day discharge after lumbar fusion. Efforts to convert from bilateral surgery have been helpful. Several reports have shown success with single interbody devices [1, 4, 6, 7, 9, 14, 16–18] and with unilateral pedicle fixation [1, 4, 5, 9, 14, 16, 18]. Two small series of minimally invasive unilateral TLIF reported mean lengths of stay of 1.6 and 2.5 days [1, 4]. Early discharge is facilitated by use of intraoperative bupivicaine, increased confidence of the surgeons and patients, transition to more stable titanium interbody devices, and more education about pain relief and other benefits of early and frequent ambulation.

We did not observe differences between the hospital stay group and the same-day discharge group in terms of validated outcomes instruments at 3 months postoperatively, other than a small difference of borderline statistical and questionable clinical significance in terms of lower leg pain (< 1 point on a 10-point VAS, p = 0.05). As our power calculations demonstrated that our sample size was adequate for the comparisons we made, the absence of differences should not be attributed to insufficient statistical power. Large series of similar surgery have used different outcome instruments but have shown agreement in terms of improvement in function and pain [16, 18]. While we reported outcome data only out to 3 months, this period appears to be most relevant when evaluating results of minimally invasive TLIF. A recent detailed review of the literature [13] reported several studies comparing minimally invasive to open TLIF and the advantages of minimally invasive procedures were seen at the time of surgery and for only a few weeks after surgery. Results at later times usually were similar between groups.

Early medical complications and readmissions within 2 weeks of surgery were not different for those younger than 65 years. A surprise finding was higher complication and readmission rates for patients 65 years or older who stayed in hospital only one night compared to patients 65 years or older with same-day discharge (Table 6). The mean age of these groups was similar and medical comorbidities were higher in the same-day discharge group. Patients 65 years or older who stayed one night in hospital had higher self-rated disability and slightly higher pain scores, which suggests they felt unable to rapidly resume activities such as ambulation. Inactivity is thought to play a major role in the postoperative complications ileus and pneumonia. Rates of readmission for medical problems have not been reported for unilateral TLIF. Reoperation rates have not been reported for unilateral TLIF, but rates after lumbar fusion have been as high as 25% [2]. There were no urgent readmissions for surgical or medical problems within 2 weeks of index surgery in this series of 1114 minimally invasive unilateral TLIF procedures.

We found that, in the setting of a high-volume spine practice, with sufficient support, surgeons experienced in minimally invasive spine surgery can consider same-day discharge for patients having minimally invasive unilateral TLIF procedures and can expect not to see an untoward increase in complications or readmissions or compromise to short-term patient-derived outcome scores. Studies in other settings are called for to validate these results.
